# Computational and biological studies of novel thiazolyl coumarin derivatives synthesized through Suzuki coupling

**DOI:** 10.3906/kim-2005-19

**Published:** 2020-12-16

**Authors:** Shaista PARVEEN, Saima KALSOOM, Rifhat BIBI, Ambreen ASGHAR, Abdul HAMEED, Waqar AHMED, Abbas HASSAN

**Affiliations:** 1 Department of Chemistry, Quaid-i-Azam University, Islamabad Pakistan; 2 SA-Centre for Interdisciplinary Research for Basic and Applied Sciences, International Islamic University, Islamabad Pakistan

**Keywords:** Coumarin thiazole, Suzuki cross-coupling, molecular dynamics simulation, antibacterial activity, antifungal activity, binding free energy calculation

## Abstract

The current investigation presents the synthesis, computational molecular-docking and biological activity studies of arylated thiazole coumarins. Aryl substituted thiazolyl coumarin derivatives were synthesized via Suzuki cross-coupling reaction. A detailed reaction condition optimization revealed that the Pd-PEPPSI-IPent precatalyst in only 2 mol% loading resulted in the desired product with high yield. The aim of this study was to examine the antimicrobial behavior of thiazole coumarin derivatives through in vitro and in silico studies. All the compounds showed activity against both antibacterial strains, Staphylococcus aureus and Escherichia coli, except **5d** . Similarly, the compounds **5a** , **5b** , and **5d** were found to be active against Trichoderma harzianum. The compound **5d** of this series was found to have a higher activity with MIC 125 mg/ml against Trichoderma harzianum. Molecular studies showed the high activities of these compounds are due to the presence of strong H-bonding and π-π interaction with their respective targets. A good correlation was observed between computational and in vitro studies.

## 1. Introduction

Antibiotic resistance is one of the leading public health concerns of the current age [1, 2], mainly due to the emergence, spread, and persistence of multidrug-resistant (MDR) bacteria or ‘superbugs’ which cannot be treated with conventional remedies. The plausible causes of the antimicrobial resistance are the widespread use of antibiotics and the weary antibiotic research progress [3]. Similarly, the incidence of systemic fungal infection has become an important complication and a major cause of morbidity and mortality in immune compromised individuals, such as those who are receiving anticancer chemotherapy, those who have had organ transplants, and AIDS patients [4,5]. In many antimicrobial drugs, the heterocyclic thiazole nucleus are an integral part of the drug as pharmacophore [6,7]. Drug designing techniques have led to the designing of novel coumarin scaffold with various bioactive entities in order to realize pharmacophores with good pharmacology as well as enhanced pharmacokinetics [8–10]. A large number of studies have been conducted on the combined effect of thiazole and coumarin scaffolds that show improved biological activities [11–14]. The present study is based on in vitro antibacterial and antifungal screening of small drug-like thiazole coumarin derivatives.

The advent of computational tools offers a rapid and complementary role in understanding the binding and inhibition patterns of different drug-like molecules against a variety of targets [15,16]. Particularly, molecular docking analysis helps to show the binding ability of ligands with desired targets [17–20]. The inhibitory potential of thiazole coumarin derivatives has been explored herein using molecular docking to explain their inhibition activities against both bacterial and fungal strains.

The basic core structure having thiazole and coumarin subunits has been synthesized through Knoevenagel condensation [21], Pechmann reaction [22], and Hantzsch reaction [23], using readily available starting materials. Suzuki cross-coupling reaction of organoboron compounds and organic halides is one of the most efficient methods for the construction of carbon-carbon bonds [24]. Overall, the commercial availability and bench top stability of the starting materials, mild reaction conditions, and the tolerance of a broad range of functionalities have extended the scope of the reaction. The NHC bound family of palladium precatalysts, including Pd-PEPPSI-IMes, Pd-PEPPSI-IPr, and Pd PEPPSI-IPent, serve as complementary; and in most cases, superior catalyst system has been widely utilized for a variety of cross coupling reactions [25,26]. Herein, we report the computational docking and biological studies of the thiazolyl coumarin derivatives synthesized through a facile Suzuki reaction.

## 2. Materials and methods

### 2.1. Chemical synthesis of the compounds

#### 2.1.1. General experimental conditions

Chemicals were purchased from Merck KGaA (Darmstadt, Germay) and Sigma-Aldrich Corp. (St. Louis, MO, USA). IR spectra were recorded on Schimadzu Fourier transform infrared spectrophotometer model 270 (Shimadzu Corporation, Kyoto, Japan), using ATR (attenuated total reflectance) facility. All the reactions were monitored by Thin-layer chromatography (TLC) using the precoated silica gel-60 F254 purchased from Merck KGaA. Flash column chromatography was carried out with silica gel as stationary phase (particle size 200–300 mesh). High resolution mass spectrometric (HRMS) experiments were carried out on Finnigan MAT-311A (Finnigan MAT GmbH, Bremen, Germany) mass spectrometer with (ESI) ionization techniques. NMR spectra were obtained using a Bruker Avance 300 MHz spectrometer (Bruker BioSpin Corp., Billerica, MA, USA) in deuterated chloroform using TMS as internal reference, at 300 MHz ( ^1^ H NMR) and 75 MHz ( ^13^ C NMR). Chemical shifts are mentioned in delta (δ) units while coupling constants (J) values are in Hertz unit (Hz).

#### 2.1.2. Synthesis of 6-bromo-3-(2-methylthiazol-4-yl)-2H-chromen-2-one (4)

The compound 4 was synthesized in slightly modified method already reported in the literature [27]. The overall synthesis consists of the following 3 steps:

In a 250 mL 2-neck round bottom flask, 5-bromosalicyladehyde (2 g, 9.95 mmol), ethyl acetoacetate (1.9 mL, 14.85 mmol), and piperidine (9 μL, 1.00 mmol) were added along with ethanol as solvent and the mixture was heated at 50 °C for 8 h. Water (20 mL) was added to quench the reaction, and the organic layer was separated. The aqueous layer was extracted with ethyl acetate (20 mL × 3). The combined organic layer was dried using sodium sulphate and concentrated under vacuum. The product 2 was obtained as yellow solid (2.18 g) in 82% yield.

3-Acetyl-6-bromo-2 *H* -chromen-2-one 2 (1.5 g, 5.62 mmol) in dichloromethane (28 mL, 0.2 M) was added to the mixture in the 2-neck round bottom flask in an ice bath. Bromine (0.15 mL, 5.62 mmol) was added dropwise, and the reaction was allowed to cool to ambient temperature overnight. Water (25 mL) was added to quench the reaction and extracted with dichloromethane (30 mL × 3). The combined organic layer was concentrated in vacuo to obtain compound 3 as yellow solid (1.63 g) in 84% yield.

A 2-neck round bottom flask was filled with 6-bromo-3-(2-bromoacetyl)-2 *H* -chromen-2-one 3 (1 g, 2.89 mmol) and thioacetamide (326.0 mg, 4.34 mmol) in ethanol (15 mL, 0.2 M). The reaction mixture was heated at 60 °C for 16 h, and the progress of the reaction was monitored by TLC. After the completion of the reaction, the solvent was evaporated in vacuo, and the crude product was purified by flash chromatography (n-hexane: ethyl acetate 20:1). The compound 4 was obtained as yellow solid in 80% yield (745 mg).

Physical data of 4:

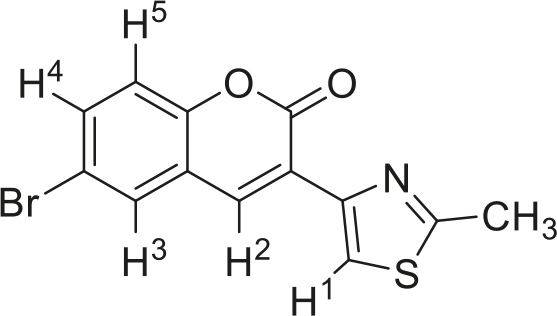



Rf = 0.5 (n-Hex: EtOAc, 9:1)

^1^ H NMR (300 MHz, CDCl _3_ , δ ppm): 8.61 (s, 1H, H ^2^ ), 8.31 (s, 1H, H1), 7.72 (d, 1H, *J* = 2.4 Hz, H ^3^ ), 7.63 (d, 1H, *J* = 8.7 Hz, H ^4^ ), 7.24 (d, 1H, *J* = 8.6 Hz, H5), 2.77 (s, 3H, CH ^3^ )

^13^ CNMR (75 MHz, CDCl _3_ , δ ppm): 165.1, 158.6, 151.8, 146.9, 137.7, 134.2, 130.5, 122.0, 121.1, 120.4, 119.2, 118.1, 18.3.

HRMS-ESI (m/z): Calcd. for C _13_ H _9_ BrNO _2_ S, [M+H] +:321.9532, found 321.9537.

FTIR (neat): ῡ (cm–1) = 3076, 1693 (C=O), 1632, 1593, 1489, 1370, 1182, 1091, 961.

#### 2.1.3. General procedure for Suzuki coupling

An oven dried 100 × 13 mm sealed tube having magnetic bead was charged with thaizole-coumarin substrate (4, 100 mg, 0.31 mmol), boronic acid (0.46 mmol), KOtBu (70 mg, 0.62 mmol) and Pd-PEPPSI-IPent precatalyst (4.8 mg, 0.006 mmol) in toluene (1.5 mL, 0.2 M). The reaction was purged with nitrogen to develop an inert atmosphere. The reaction was carried out at 80 °C for 24 h. The reaction mixture was allowed to cool at room temperature and loaded onto silica gel, purified via flash column chromatography (SiO _2_ , using solvent system of n-Hexane and ethyl acetate in ratio of 9:1), and afforded the desired products 5a–5e in good to excellent yields.

#### 2.1.4. Characterization data of 5a–5e:

**3-(2-methylthiazol-4-yl)-6-phenyl-2H-chromen-2-one (5a)**

The reaction was set up with phenylboronic acid (56 mg, 0.46 mmol). The product 5a was obtained as white solid in 90% yield (89 mg).

M.P. 192–194 oC, Rf = 0.52, (n-Hex: EtOAc, 9:1)

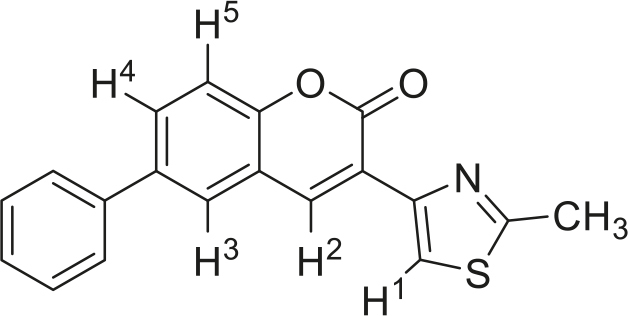



^1^ H NMR(300 MHz, CDCl _3_ , δ ppm): 8.67 (s, 1H, H ^2^ ), 8.34 (s, 1H, thiazole H1), 7.83 (d, 1H, *J* = 2.1 Hz, H ^3^ ), 7.78 (dd, 1H, *J* = 8.7, 2.1 Hz, H ^4^ ), 7.63 (d, 1H, *J* = 8.7 Hz, H5) 7.39–7.53 (m, 5H, phenyl), 2.80 (s, 3H, thiazole-CH ^3^ ).

^13^ C NMR (75 MHz, CDCl _3_ , δ ppm): 165.8, 159.8, 151.8, 146.9, 139.5, 137.8, 134.2, 130.5, 129.1, 127.8, 127.1, 121.9, 121.1, 120.4, 118.1, 117.2, 19.2.

HRMS-ESI (m/z): Calcd. for C19H14NO2S, [M+H] ^+^ : 320.0740, found 320.0746.

FTIR (neat): ῡ (cm–1) = 3086, 1688(C=O), 1650, 1541, 1452, 1365, 1133, 1092, 963.

**3-(2-methylthiazol-4-yl)-6-o-tolyl-2H-chromen-2-one (5b)**

The reaction was set up with *o* -tolylboronic acid (63 mg, 0.46 mmol). The product 5b was obtained as white solid in 94% yield (97 mg).

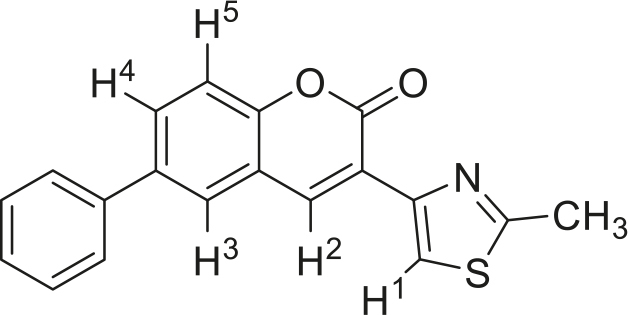



M.P. 192–195 oC, Rf = 0.53, (n-Hex: EtOAc, 9:1)

^1^ H NMR (300 MHz, CDCl _3_ , δ ppm): 8.76 (s, 1H, H ^2^ ), 8.35 (s, 1H, thiazole H1), 7.58 (d, 1H, J = 1.8 Hz, H ^3^ ), 7.52 (dd, 1H, J = 1.8, 8.7 Hz, H ^4^ ), 7.43 (d, 1H, J = 8.7 Hz, H5), 7.24–7.33 (m, 4H-phenyl), 2.80 (s, 3H, thiazole-CH ^3^ ), 2.31 (s, 3H, phenyl-CH ^3^ ).

^13^ C NMR (75 MHz, CDCl _3_ , δ ppm): 165.5, 159.8, 152.0, 147.5, 140.1, 139.2, 138.5, 135.4, 132.6, 130.6, 129.8, 128.6, 127.9, 126.1, 121.2, 119.8, 119.2, 116.1, 20.5, 19.2.

HRMS-ESI (m/z): Calcd. for C20H16NO2S, [M+H] ^+^ : 334.0896, found 334.0898.

FTIR (neat): ῡ (cm–1) = 3081, 1681 (C=O), 1649, 1553, 1457, 1368, 1165, 1088, 954.

**3-(2-methylthiazol-4-yl)-6-m-tolyl-2H-chromen-2-one (5c)**

The reaction was set up with *m* -tolylboronic acid (63 mg, 0.46 mmol). The product 5c was obtained as white solid in 85% yield (88 mg).

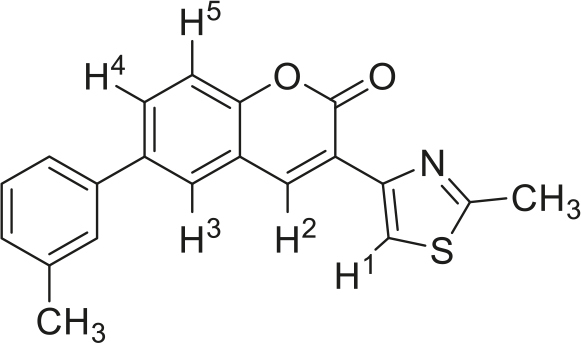



M.P. 196–197 °C, Rf = 0.53, (n-Hex: EtOAc, 9:1)

^1^ H NMR (300 MHz, CDCl _3_ , δ ppm): 8.84 (s, 1H, H ^2^ ), 8.35 (s, 1H, thiazole H1), 7.81 (d, 1H, *J* = 2.1 Hz, H ^3^ ), 7.77 (dd, 1H, *J* = 2.4, 8.4 Hz, H ^4^ ), 7.23(d, 1H, *J* = 8.4 Hz, H5), 7.22–7.45 (m, 4H, H-phenyl), 2.82 (s, 3H, thiazole-CH ^3^ ), 2.46 (s, 3H, phenyl-CH ^3^ ).

^13^ C NMR(75 MHz, CDCl _3_ , δ ppm): 165.4, 159.7, 152.5, 150.0, 145.4, 139.3, 138.3, 136.5, 132.8, 130.3, 129.9, 128.4, 127.8, 127.4, 126.4, 122.3, 121.6, 116.3, 22.3, 19.4.

HRMS-ESI (m/z): Calcd. for C _20_ H _16_ NO _2_ S, [M+H] ^+^ : 334.0896, found 334.0899.

FTIR (neat): ῡ (cm–1) = 3087, 1691 (C=O), 1646, 1578, 1489, 1371, 1172, 1091, 958.

**6-(4-methoxyphenyl)-3-(2-methylthiazol-4-yl)-2H-chromen-2-one (5d)**

The reaction was set up with p-methoxyphenylboronic acid (70 mg, 0.46 mmol). The product 5d was obtained as white solid in 94% yield (99 mg).

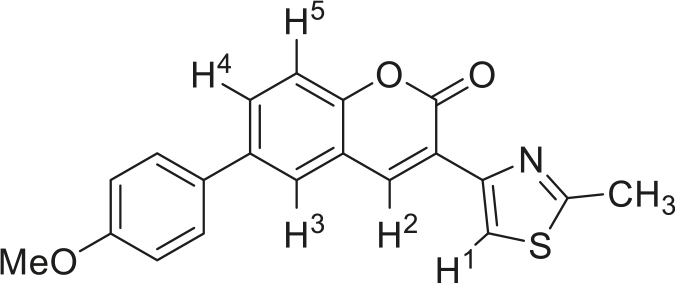



M.P. 194–198 oC, Rf = 0.51, (n-Hex: EtOAc, 9:1)

^1^ H NMR (300 MHz, CDCl _3_ , δ ppm): 8.82 (s, 1H, H ^2^ ), 8.35 (s, 1H, thiazole H1), 7.77 (d, 1H, J = 2.1 Hz, H ^3^ ), 7.74 (dd, 1H, J = 2.1, 8.7 Hz, H ^4^ ), 7.56 (d, 2H, J = 8.7 Hz, phenyl H-2,6), 7.43 (d, 1H, J = 8.4 Hz, H5), 7.03 (d, 2H, J = 8.7 Hz, phenyl H-3,5), 3.89 (s, 3H, methoxy), 2.82 (s, 3H, thiazole-CH ^3^ ).

^13^ C NMR (75 MHz, CDCl _3_ , δ ppm): 164.3, 158.5, 149.7, 146.5, 139.1, 136.7, 134.1, 129.8, 128.9, 127.1, 126.8, 120.8, 120.1, 119.5, 119.1, 58.7, 19.3.

HRMS-ESI (m/z): Calcd. for C _20_ H _16_ NO _3_ S [M+H] ^+^ : 350.0845, found 350.0847.

FTIR (neat): ῡ (cm–1) = 3084, 1686 (C=O), 1642, 1573, 1484, 1372, 1178, 1096, 951.

**3-(2-methylthiazol-4-yl)-6-(naphthalen-1-yl)-2H-chromen-2-one (5e)**

The reaction was set up with 1-naphthylboronic acid (79 mg, 0.46 mmol). The product 5e was obtained as white solid in 88% yield (101 mg).

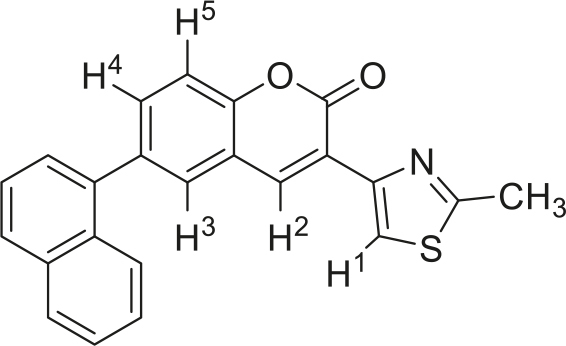



M.P. 199–194 oC, Rf = 0.53, (n-Hex: EtOAc, 9:1)

^1^ H NMR (300 MHz, CDCl _3_ , δ ppm): 8.79 (s, 1H, H ^2^ ), 8.37 (s, 1H, thiazole H1), 7.75 (d, 1H, *J* = 1.5 Hz, H ^3^ ), 7.68 (dd, 1H, *J* = 1.8, 8.4 Hz, H ^4^ ), 7.97–7.45 (m, 8H), 2.80 (s, 3H, thiazole-CH ^3^ ).

^13^ C NMR (75 MHz, CDCl _3_ , δ ppm): 165.5, 159.9, 152.3, 147.4, 139.3, 138.3, 137.4, 133.8, 133.4, 129.4, 128.5, 128.3, 127.2, 126.5, 126.1, 125.5, 125.4, 121.3, 119.8, 116.3, 19.2.

HRMS-ESI (m/z): Calcd. for C _23_ H _16_ NO _2_ S [M+H] ^+^ : 370.0896, found 370.0900.

FTIR (neat): ῡ (cm–1) = 3091, 1679 (C=O), 1644, 1579, 1493, 1365, 1191, 1078, 939.

### 2.2. Biological activities of the compounds

#### 2.2.1. Antibacterial assay

A strain of gram-positive bacteria Staphylococcus aureus (ATCC: 6538) and gram-negative bacteria Escherichia coli (ATCC: 8739) were obtained from the culture collections of SA-Centre for Interdisciplinary Research in Basic Science (SA-CIRBS), IIUI. Both cultures were refreshed in nutrient broth. The bacterial culture was grown in nutrient agar to obtain single colonies of both strains. After culturing and refreshing, antibacterial activity was performed via agar well diffusion method. The media was prepared by suspending 2.8 gm of nutrient agar powder in 100 mL of distilled water in a flask. The media was autoclaved at 121 °C for 15 min; afterwards, it was left to cool but not solidify. About 20 mL of the media was poured into each petri dish in a laminar flow hood. The petri dishes were left on the sterile surface until the media solidified. Lids were placed on each petri dish. Bacterial dilution in saline solution was prepared according to McFarland standard. The agar plate surface was inoculated by pouring 100 µL of bacterial suspension over the entire agar surface and spreading it with glass rod [28]. The plates were left for drying. A 6 to 8 mm-diameter hole was punched aseptically with a sterile cork borer, and 20 µL of each compound was introduced into the well. DMSO (dimethyl sulfoxide) and PenStrep were used as negative and positive control. The agar plates were incubated at 25 °C for 24 h. The antimicrobial agent diffused in the agar medium and inhibited the growth of microbes. In initial step, qualitative test was performed to find out active compounds, followed by minimum inhibitory concentration (MIC) was performed on the active compounds. The concentration for MIC includes 500, 250, 125, 62.5, and 31.25 µg/mL[29].

#### 2.2.2. Antifungal assay

The fungal strain Trichoderma harzianum was obtained from culture collections of SA-Centre for Interdisciplinary Research in Basic Science (SA CIRBS), IIUI. Single spore technique was used for Trichoderma harzianum to get a pure culture. The culture was refreshed in potato dextrose agar (PDA). Antifungal activity was performed via agar well diffusion method. The PDA plate surface was inoculated by pouring 100 µL of fungal suspension over the entire agar surface and spreading it with a glass rod. The fungal suspension was prepared by mixing fungal spores in autoclaved distilled water. The plates were left to dry. Then, a hole with a diameter of 6 to 8 mm was punched aseptically with a sterile cork borer, and 20 µL of each compound was introduced into the well. DMSO and fluconazole were used as positive and negative control. The agar plates were incubated at 25 °C for 4 days. Similar to the antibacterial assay, a qualitative test was also performed in antifungal activity to find out active compounds, and then minimum inhibitory concentration (MIC) was carried out on active compounds against Trichoderma harzianum to find out the lowest concentration of compound that inhibits the growth of fungus. Serial dilution of compounds was performed. The concentration for MIC includes 500, 250, 125, 62.5, and 31.25 µg/mL.

#### 2.2.3. Molecular docking studies

The 3D structures of antibacterial target (PDB id 1YLJ) [30] and antifungal target (PDB id 2YOK) were fetched from Protein Data Bank Research Collaboratory for Structural Bioinformatics (PDB) (2020). Protein Data Bank [online]. Website u1eb6 [accessed 00 Month Year]., and all the atoms were prepared, charged, protonated, and minimized via MOE 2016 suite. The chemical structures of the synthesized compounds were built and saved in their 3D conformations by Builder tool incorporated in MOE 2016. The following factors were used for energy minimization gradient: 0.05, Force Field: MMFF94X+Solvation, Chiral Constraint: Current Geometry. Energy minimization was terminated when the root mean square gradient fell below 0.005. The minimized structures of the complexes were used as the template for molecular docking. Ten conformations were generated for each docked ligand-target complex. Lowest energy minimized conformation of each docked complex was used for further analysis.

## 3. Results and discussion

### 3.1. Chemical synthesis

**Scheme 1:**
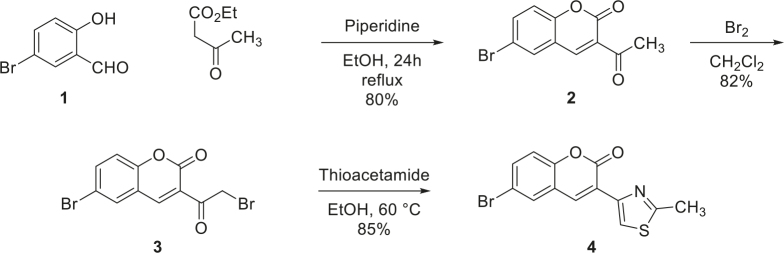
The synthesis of thiazole coumarin derivative 4.

The thiazole-coumarin substrate was prepared in 3 steps following a slightly modified reported procedure[14]. In the first step, a Knoevenagel condensation using 5-bromosalicylaldehyde and ethyl acetoacetate was performed in the presence of catalytic amount of piperidine to obtain 3-acetyl-6-bromo-2 *H* -chromen-2-one (2) in 80% isolated yield. In the second step, the intermediate was further treated with bromine in dichloromethane to obtain the monobromination product 3 in 82% yield. Finally, the Hantzch thiazole synthesis of 3 with thioacetamide formed the thiazole coumarin product 4 having bromo substituent at 6-position in 85% isolated yield (Scheme 1).

Suzuki coupling is one of the most efficient and widely used methods for the C-C coupling, yet there are some shortcomings associated with the reaction. The scramble product formation, and catalyst decomposition may occur during the course of reaction. In addition, the associated homo coupled by-product can diminish the desire product and may hamper the isolation of the pure product. To mitigate these problems, an exhaustive reaction condition optimization maybe required to get the coupled product in good yield. The screening of reaction conditions was performed, and the results are given in Table 1. Initially, the coupling of thiazolyl substituted 6-bromocoumarin (4) and phenylboronic acid in the presence of palladium acetate, triphenylphosphine, and potassium carbonate in toluene as a solvent provided the desired product 5a in only 22% isolated yield (entry 1). During the solvent screening, change of solvent from toluene to THF could not improve the yield (entry 2). The study of the ligands revealed that utilizing JohnPhos as the ligand resulted in a moderately improved 38% yield, while pre-formed PdCl2dppf catalyst provided a similar yield (entries 3-4). The sterically demanding Buchwald ligands can withstand the test of time for variety of coupling reactions [31], and the bidentate ferrocene based DPPF ligand favors the reductive elimination during the catalytic cycle. Both of the above ligands have shown some improvement in the coupling reaction. The strong sigma donor N-heterocyclic carbene (NHC) based Pd-PEPPSI family of precatalyst systems have been developed by Prof. Michael G. Organ, and these have shown eclectic utility and robustness [32]. Therefore, employing precatalyst Pd-PEPPSI-IPr under otherwise similar conditions resulted in 25% desired product (entry 5). When the reaction was performed by switching the base to KtOBu, the desired 5a was formed with enhanced yield (entry 6). To our delight, when the reaction was performed with more sterically demanding Pd-PEPPSI-IPent catalyst in the presence of KtOBu in toluene, the product 5a was obtained in 95% yield (entry 7). At this point, when the reaction was further tested with NatOBu or with higher loading of KtOBu, the yield could not be improved (entries 8-9). Lowering the Pd-PEPPSI-IPent catalyst loading upto 2 mol%, the yield of the desired product was 90% (entry 10). Thus increasing or decreasing the base loading or changing the type of base could not improve the yield. As previous studies of PEPPSI precatalyst explored the importance of sterics in the backbone of the NHC core in the cross coupling reactions, which prompted us to prompted to optimize the steric bulk of NHC core of the Pd-PEPPSI precatalyst. The optimizations (Table 1) showed that a moderate yield of the final coupled product was obtained with Pd-PEPPSI-IPr, while a quantitative yield was obtained when Pd-PEPPSI-IPent was used. Thus, the suitable catalyst for the current Suzuki cross coupling of thiazolyl coumarin system was found to be Pd-PEPPSI-IPent.

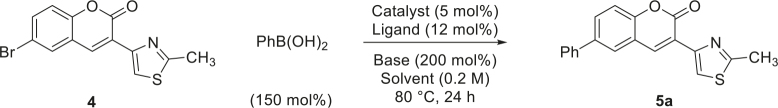



**Table 1:** Reaction condition optimization for Suzuki coupling of thiazole coumarin derivative and the structure of the catalyst and liganda.

S. No.	Pd Catalyst	Ligand	Base	Solvent	Yield%
1.	Pd(OAc)2	PPH ^3^	K2CO3	Toluene	22
2.	Pd(OAc)2	PPH ^3^	K2CO3	THF	22
3.	Pd(OAc)2	JohnPhos	K2CO3	Toluene	38
4.	PdCl2dppf	-	K2CO3	Toluene	35
5.	Pd-PEPPSI-IPr	-	K2CO3	Toluene	25
6.	Pd-PEPPSI-IPr	-	KOtBu	Toluene	46
7.	Pd-PEPPSI-IPent	-	KOtBu	Toluene	95
8.	Pd-PEPPSI-IPent	-	NaOtBu	Toluene	32
9.	Pd-PEPPSI-IPent	-	bKOtBu	Toluene	33
10.	cPd-PEPPSI-IPent	-	KOtBu	Toluene	90

aReaction conditions: thiazole coumarin (4, 0.31 mmol, 100 mol%), boronic acid (0.46 mmol, 150 mol%), base (0.62 mmol, 200 mol %,), Pd-catalyst (0.016 mmol, 5 mol%), ligand (0.037 mmol, 12 mol%), toluene (0.2 M), 80 °C, 24 h. bKOtBu (0.93 mmol, 300 mol%), cPd-PEPPSI-IPent (0.006 mmol, 2 mol%) was used.

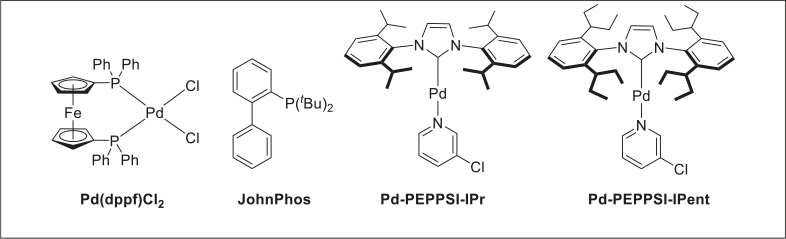



Various types of boronic acids were utilized to extend the scope of reaction. Structurally variable arylated thiazole coumarins were synthesized as per optimized reaction conditions, as shown in Table 2. Alkyl, alkoxy, and aryl-substituted boronic acids were employed in the reaction, leading to the formation of the final arylated product in excellent yields. o-tolylboronic acid was reacted under the optimized conditions, yielding the desired product 5b in 94% isolated yield, while m-tolylboronic acids resulted in the final coupled product 5c in 85% yield. Similarly, 4-methoxypenylboronic acid resulted in the end product 5d in 91% yield. 1-Naphthylboronic acid also provided the comparable yield of 88% of the desired product 5e.

**Table 2:** Derivatization of thiazolyl coumarin substrate with different boronic acids.

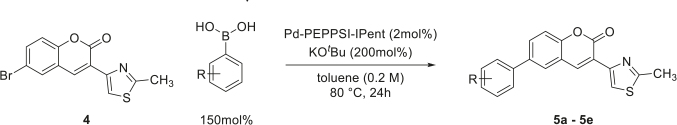
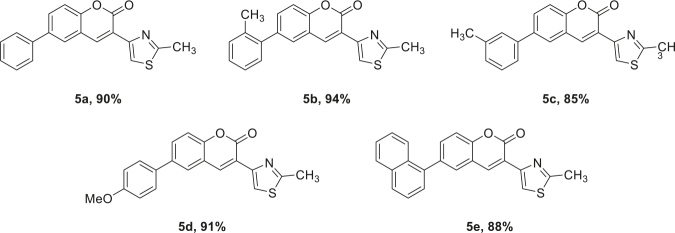

### 3.2. Biological evaluation of the compounds

#### 3.2.1. Antibacterial assay

Qualitative test was performed to screen active compounds against a strain of gram-positive bacteria Staphylococcus aureus (ATCC: 6538) and a strain of gram-negative bacteria Escherichia coli (ATCC: 8739).

All the compounds showed activity against both strains except 5d. PenStrep and DMSO were used as positive control and negative control respectively. Compound 5a showed higher inhibition against both gram-positive and gram-negative bacteria (Table 3).

**Table 3:** Zone of inhibition of thiazole coumarin derivatives against 2 strains of bacteria.

S. No.	Compounds	Zone of inhibition (mm)	
		Staphylococcus aureus	Escherichia coli
1	5a	15	17
2	5b	13	16
3	5c	15	13
4	5d	-	-
5	5e	11	11

The minimum inhibitory concentration (MIC) of 5a, 5b, 5c, and 5e was determined by agar well diffusion method. The concentration of the compounds ranges from 31.25 to 500 µg/mL. The MIC value of 5a was determined to be 62.5 µg/mL against both gram-positive and gram-negative bacteria. The compound 5b exhibited MIC at 500 µg/mL against Staphylococcus aureus and 125 µg/mL against Escherichia coli (Tables 4 and 5).

**Table 4:** Minimum inhibitory concentration of active compounds against Staphylococcus aureus (+).

S. No.	Compounds	Concentrations (µg/mL)				
		500	250	125	62.5	31.25
		Zone of inhibitions (mm)				
1	5a	13	11	10	7	-
2	5b	10	-	-	-	-
3	5c	12	9	-	-	-
4	5e	8	-	-	-	-

**Table 5:** Minimum inhibitory concentration of active compounds against Escherichia coli (–).

S. No.	Compounds	Concentrations (µg/mL)				
		500	250	125	62.5	31.25
		Zone of inhibitions (mm)				
1	5a	15	12	10	9	-
2	5b	14	13	10	-	-
3	5c	8	-	-	-	-
4	5e	9	-	-	-	-

#### 3.2.2. Antifungal assay

The synthesized compounds were also tested on strain of Trichoderma harzianum on PDA plates. Concentration of the compounds used was 1 mg/mL in DMSO. Three out of 5 compounds showed inhibition of Trichoderma harzianum. Miltefosine and DMSO were used as positive control and negative control respectively. Compound 5d was found to be more active against Trichoderma harzianum (Table 6).

**Table 6:** Zone of inhibition of thiazole coumarin derivatives against Trichoderma harzianum.

S. No.	Compounds	Zone of inhibition (mm)
		Trichoderma harzianum.
1	5a	11
2	5b	13
3	5c	-
4	5d	14
5	5e	-
6	Miltefosine	17

The MIC of synthesized compounds was determined by agar well diffusion method. Compounds 5a, 5b, and 5d were tested for their MIC value. Only compound 5d showed MIC value at concentration 125 µg/mL against Trichoderma harzianum (Table 7).

**Table 7:** MIC of active compound against Trichoderma harzianum.

S. No.	Compounds	Concentrations (µg/mL)				
		500	250	125	62.5	31.25
		Zone of inhibitions (mm)				
1	5d	13	11	9	-	-

#### 3.2.3. Docking study of bacterial protein

Bacterial protein PDB ID 1YLJ was downloaded from Protein Data Bank and prepared in molecular operating environment (MOE) for docking. Ligand was removed, and mdb file of compounds were docked in place of ligand. The results are mentioned in Table 8. As stated in antibacterial activity section, compounds 5a and 5b were found to be the most active derivatives against Staphylococcus aureus (ATCC: 6538) and gram-negative bacteria Escherichia coli (ATCC: 8739).

**Table 8:** Hydrophobic and hydrophilic interactions of docked compounds in active site of 1YLJ.

Sr. No.	Compounds	H-bonding		π-π interaction	Binding energy(KJ/mol)	ZI (mm)Escherichia coli
		Distance (Å)	Amino acids	Amino acids		
1.	5a	1.82	Thr216	Lys234	–10.12	17
2.	5b	2.15, 3.19 2.27	Ser130, Lys73 Ser70	-	–10.34	16
3.	5c	2.56	Ser70	Arg276	–10.91	13
4.	5d	-	-	-	–10.34	-
5.	5e	2.11	Thr216	Lys234	-–10.65	11
6.	Penicillin	2.19, 2.63	Ser130, Ser70	Arg276	–11.32	28

**Figure 1:**
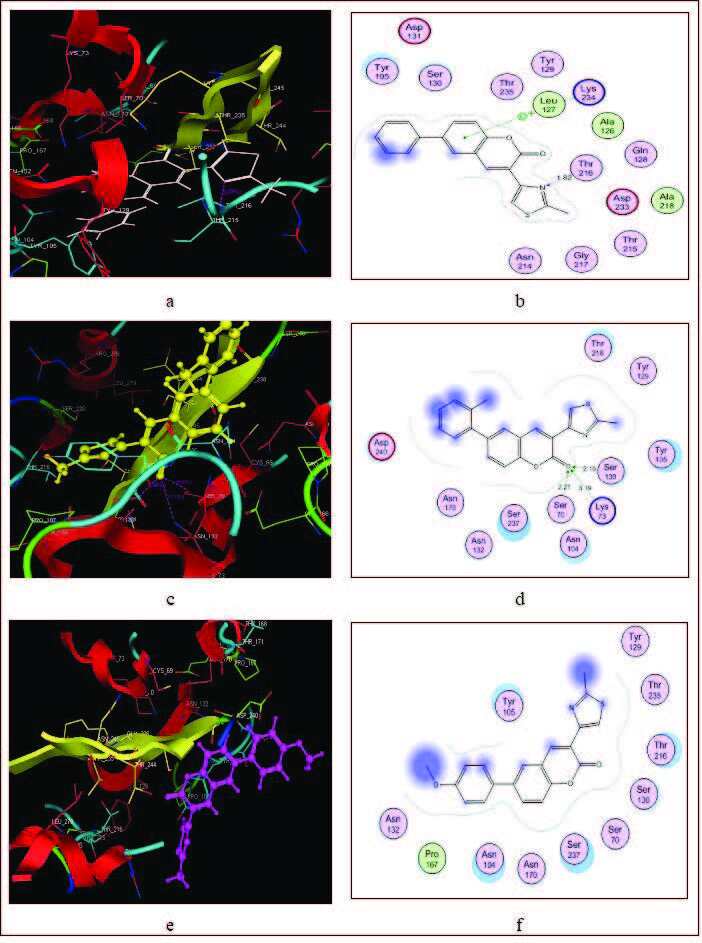
3D & 2D docked poses of compounds of the target 1YLJ. a) 5a docked in the active site, b) 5a among the key amino acid residues of the protein, c) 5b docked in the active site, d) 5b among the key amino acid residues of the protein, e) 5d docked in the active site, f) 5d among the key amino acid residues of the protein.

The main purpose of docking studies was to investigate the possible interaction of these compounds with target. Active site of target 1YLJ based on hydrophobic and hydrophilic amino acids, such as Asp131, Tyr105, Ser130, Thr235, Tyr129, Leu127, Lys234, Ala126, Thr216, Gln128, Asp233, Ala218, Thr215, Gly217, Asn214, Gly217, Thr215, Ser237, Ser70, Lys73, Ser130, and Arg276. All docked compounds showed strong hydrogen binding with Thr216, Asn214, Lys234, Thr216, Ser130, Lys73, Ser70, and Ser237, in addition to arene-π interactions with Lys234 and Arg276, as shown in Figure 1. High antibacterial behavior of compounds 5a and 5b may be due to their strong hydrophobic and hydrophilic interactions with key amino acids, as shown in Table 8. Aromatic moiety of compound 5a has arene-π interactions with Lys234, and N-atom showed hydrogen bonding with Thr216 at distance of 1.82 Å. All the thiazole coumarins compounds bind at the same place.

#### 3.2.4. Antifungal docking results

**Figure 2:**
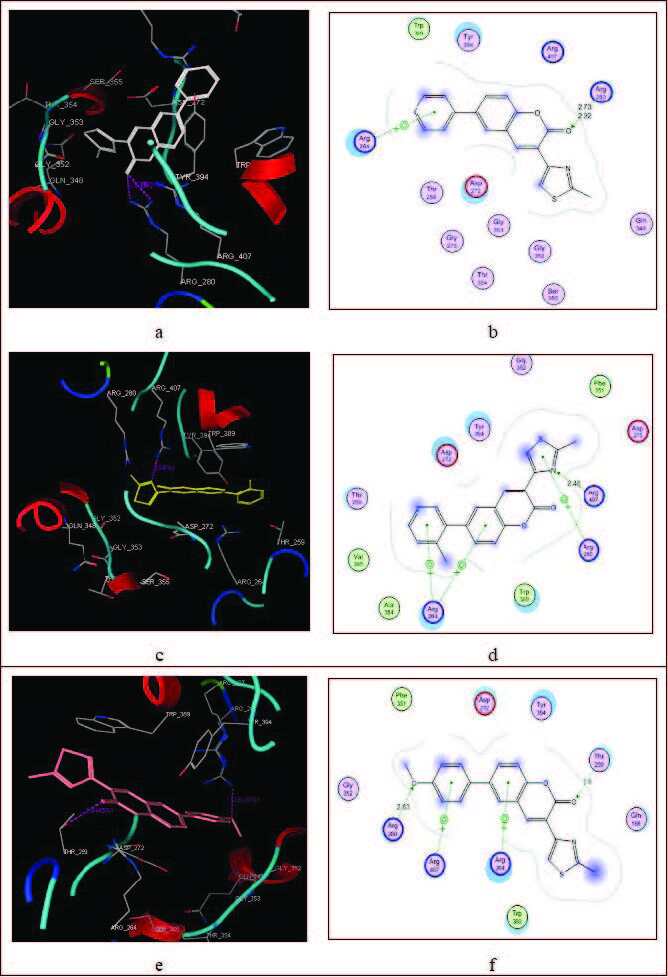
3D & 2D docked view of thiazole coumarins in active site of fungal target. Dotted lines showed the binding interactions with key amino acids. a) 5a docked in the active site, b) 5a among the key amino acid residues of the protein, c) 5b docked in the active site, d) 5b among the key amino acid residues of the protein, e) 5d docked in the active site, f) 5d among the key amino acid residues of the protein.

The results showed that the thiazole coumarin derivatives have some antifungal activity. According to antifungal screening, 3 of the synthesized compounds effectively inhibited the growth of the tested fungal strain. Compound 5d showed the best activity in inhibiting the growth of the fungal strain. In order to check the inhibition pattern of tested compound, molecular docking of compounds against target PDB id: 2YOK was performed. The high inhibitory activity of compound 5d may be attributed to its strong binding interactions with Thr259, Arg280, Arg264, and Arg407. Methoxy group at para position of ring and the carbonyl of compound showed H-binding with Arg260 and Thr259 with distances of 2.63 & 2.5 Å, respectively. Similarly, aromatic moieties of compound 5b showed arene-π interactions with Arg407-Arg264. Compound 5c and 5e did not show any type of interactions with key residues, as shown in Table 9. Activity of compounds 5a, 5b, and 5d is due to their strong hydrophobic and hydrophilic interactions with key residues, as shown in Figure 2.

**Table 9:** Dock binding interactions of antifungal drugs with PDB id: 2YOK.

Sr. No.	Compounds	H-bonding		π-π interact.	Binding energy(KJ/mol)	ZI (mm)Trichoderma harzianum
		Distance (Å)	Amino acids	Amino Acids		
1.	5a	2.73	Arg280	Arg264	–10.42	11
2.	5b	2.46	Arg407	Arg280, Arg264	–10.39	13
3.	5c	-	-	-	–10.87	-
4.	5d	2.5, 2.63	Thr259, Arg280	Arg264, Arg407	–11.34	14
5.	5e	-	-	-	–10.67	-
6.	Miltefosine	2.65,2.122.15,3.11	Gly A256, Arg A280, Arg A350, Pro A103	-	–11.37	17

## 4. Conclusion

Pd-PEPPSI-catalyzed Suzuki coupling strategy was utilized to cross couple thiazole-coumarin based coumarin derivatives with array of substituted aryl boronic acid to synthesize arylated thiazole coumarins in excellent yield using mild reaction conditions. Molecular docking studies were performed to evaluate the drug-like potential of the synthesized moieties. These studies were performed to investigate the interaction modes between the compounds and active site of antibacterial and antifungal drug targets. A good correlation was observed between in vitro and in silico studies. It can be inferred that the synthesized thiazole coumarin derivatives are useful to assess in further studies to develop new antimicrobial agents with higher efficacy.
